# High-Resolution Comparative Genomic Hybridization of Inflammatory Breast Cancer and Identification of Candidate Genes

**DOI:** 10.1371/journal.pone.0016950

**Published:** 2011-02-09

**Authors:** Ismahane Bekhouche, Pascal Finetti, José Adelaïde, Anthony Ferrari, Carole Tarpin, Emmanuelle Charafe-Jauffret, Colette Charpin, Gilles Houvenaeghel, Jocelyne Jacquemier, Ghislain Bidaut, Daniel Birnbaum, Patrice Viens, Max Chaffanet, François Bertucci

**Affiliations:** 1 Marseille Cancer Research Center (CRCM), UMR891 Inserm, Institut Paoli-Calmettes (IPC), Department of Molecular Oncology, Marseille, France; 2 Department of Medical Oncology, Institut Paoli-Calmettes (IPC), Marseille, France; 3 Université de la Méditerranée, Marseille, France; 4 Department of BioPathology, Institut Paoli-Calmettes (IPC), Marseille, France; 5 Department of Pathology, Hôpital Nord, Marseille, France; 6 Department of Surgery, Institut Paoli-Calmettes (IPC), Marseille, France; 7 Bioinformatics, Marseille Cancer Research Center (CRCM), Marseille, France; University of Birmingham, United Kingdom

## Abstract

**Background:**

Inflammatory breast cancer (IBC) is an aggressive form of BC poorly defined at the molecular level. We compared the molecular portraits of 63 IBC and 134 non-IBC (nIBC) clinical samples.

**Methodology/Findings:**

Genomic imbalances of 49 IBCs and 124 nIBCs were determined using high-resolution array-comparative genomic hybridization, and mRNA expression profiles of 197 samples using whole-genome microarrays. Genomic profiles of IBCs were as heterogeneous as those of nIBCs, and globally relatively close. However, IBCs showed more frequent “complex” patterns and a higher percentage of genes with CNAs per sample. The number of altered regions was similar in both types, although some regions were altered more frequently and/or with higher amplitude in IBCs. Many genes were similarly altered in both types; however, more genes displayed recurrent amplifications in IBCs. The percentage of genes whose mRNA expression correlated with CNAs was similar in both types for the gained genes, but ∼7-fold lower in IBCs for the lost genes. Integrated analysis identified 24 potential candidate IBC-specific genes. Their combined expression accurately distinguished IBCs and nIBCS in an independent validation set, and retained an independent prognostic value in a series of 1,781 nIBCs, reinforcing the hypothesis for a link with IBC aggressiveness. Consistent with the hyperproliferative and invasive phenotype of IBC these genes are notably involved in protein translation, cell cycle, RNA processing and transcription, metabolism, and cell migration.

**Conclusions:**

Our results suggest a higher genomic instability of IBC. We established the first repertory of DNA copy number alterations in this tumor, and provided a list of genes that may contribute to its aggressiveness and represent novel therapeutic targets.

## Introduction

Inflammatory breast cancer (IBC) [1] is one of the most lethal forms of breast cancer because of its high metastatic potential [2]. IBC is classified T4d in the TNM-UICC classification. Diagnosis is based on clinical signs: edema, erythema, pain, breast widening, and induration [2]. Most cases are associated with a ductal type and a high histological grade [3]. The presence of tumor emboli in dermal lymphatics, although not mandatory for the diagnosis, is a pathological hallmark of 50–75% of IBCs, Emboli are non-adherent cell clusters that rapidly spread by continuous passive dissemination [4], thus favoring both distant metastasis and local recurrence. Despite progresses due to the multi-modality treatment [2], [5], the prognosis remains poor with a 3-year survival of ∼50% [6]. New molecular therapeutic targets need to be identified to improve treatment and increase patient survival.

Molecular mechanisms underlying IBC are poorly known (for review, see [7], [8]). IBCs are more frequently estrogen receptor (ER) and progesterone receptor (PR) negative, ERBB2 and EGFR positive. They frequently present P53 alterations and WISP3 loss-of-expression [9]–[15]. They show high angiogenic and angioinvasive capacities and express high levels of angiogenic factors [16]. They frequently overexpress RHOC [17]–[20], E-Cadherin [18], [21], [22], and NF-κB pathway-related proteins [23], [24]. Recently, a role for eIF4G1 has been suggested in the formation of tumor emboli, pointing to the importance of translation control in IBC [25].

High-throughput molecular approaches have led to important insights in the molecular heterogeneity of non-IBC (nIBC), notably by identifying biologically and clinically relevant subtypes (luminal A and B, basal, ERBB2-overexpressing, normal-like) [26]. More recently, IBCs have been studied at the mRNA level using DNA microarrays [27]–[33]. The results showed the presence of the five molecular subtypes in IBCs, and the definition of IBC *versus* nIBC gene expression signatures. But the studied series remain relatively small, with 35 IBC samples in the largest one [27], [28]. DNA copy number alterations (CNAs) may account for phenotypic and expression differences between IBCs and nIBCs. However, in contrast to nIBCs [34]–[39], genomic imbalances have not yet been analyzed in IBC by using recent techniques such as array-comparative genomic hybridization (aCGH) or SNP-arrays. The rare genomic studies performed to date used low resolution methods [40]–[42]. Similarly, whole-genome integrated studies (associating analysis of DNA CNAs and mRNA expression levels) have been done in nIBC [34], [39], [43], [44], but never in IBC. Such approaches provide opportunities to better elucidate IBC at the DNA and RNA levels.

Here, we have studied and compared DNA CNAs and mRNA expression deregulation on a whole-genome scale in a large series of IBCs and nIBCs. To our knowledge, this is both the first high-throughput aCGH analysis of IBC and the first whole-genome integrated analysis comparing IBC *vs* nIBC. This is also the largest series of IBC profiled using high-throughput molecular analyses.

## Materials and Methods

### Breast cancer samples and histoclinical characteristics

Pre-treatment tumor tissues were collected from 197 patients with invasive adenocarcinomas, who underwent surgical biopsies or initial surgery at the Institut Paoli-Calmettes (IPC, Marseille, France) between 1987 and 2007. Each patient gave written informed consent and the study was approved by the IPC “Comité d'Orientation Stratégique”. Tumor samples were macrodissected and frozen in liquid nitrogen within 30 minutes of removal. Before RNA extraction, tumor sections were reviewed by two pathologists (ECJ and JJ) and contained more than 60% of tumor cells. The 197 samples comprised 63 IBCs and 134 nIBCs. IBC tumors were selected upon clinical criteria as T4d tumor. The control group (nIBCs) represented a mixture of early (121 samples, including 68 with pathological axillary lymph node involvement).and locally-advanced stages (13 samples), as found in previous studies [27]–[29], [32], [33]. Using only locally-advanced cases as controls would introduce a bias toward the discovery of molecular differences related to the difference of growth kinetics between IBCs (sudden onset and rapid growth) and nIBCs (long-standing evolution with slower growth). Immunohistochemistry (IHC) status was available on most samples for ER (positivity cut-off: 10%), ERBB2 (0–3+ score, DAKO HercepTest, with >1+ defined as positive), and P53 (positivity cut-off: 1%). Patients were treated according to standard guidelines. The main histoclinical characteristics are listed in Table 1. As expected, IBCs were associated with more unfavorable prognostic features and poorer survival than nIBCs.

**Table 1:** Histoclinical characteristics of the 197 breast cancer samples.

Characteristics (N)	IBC	nIBC	*p*
	N = 63 (%)	N = 134 (%)	
Median age, years (range) (197)	48 (24–82)	56 (28–84)	1.26E-03
Pathological tumor size, pT (133)			
pT1	NA	31 (23%)	
pT2	NA	70 (53%)	
pT3	NA	32 (24%)	
Pathological axillary lymph node status, pN (133)			
negative	NA	57 (43%)	
positive	NA	76 (57%)	
Grade (190)			1.35E-12
1	0 (0%)	32 (24%)	
2	10 (17%)	62 (47%)	
3	48 (83%)	38 (29%)	
IHC ER status (197)			7.74E-03
negative	33 (52%)	43 (32%)	
positive	30 (48%)	91 (68%)	
IHC ERBB2 status (171)			2.16E-04
negative	30 (61%)	107 (88%)	
positive	19 (39%)	15 (12%)	
IHC P53 status (163)			5.82E-04
negative	15 (35%)	79 (66%)	
positive	28 (65%)	41 (34%)	
Molecular subtype (197)			5.08E-05
basal	13 (21%)	25 (19%)	
ERBB2	13 (21%)	10 (7%)	
luminal A	9 (14%)	63 (47%)	
luminal B	15 (24%)	18 (13%)	
normal	13 (21%)	18 (13%)	
Genomic pattern (173)			9.30E-04
complex sawtooth	16 (33%)	23 (19%)	
complex firestorm	27 (55%)	52 (42%)	
simplex	6 (12%)	49 (40%)	
5-year MFS (191)	37%	80%	4.40E-10
5-year OS (181)	57%	84%	5.50E-11
NA, not assessable			

### DNA and RNA extraction

DNA and RNA were extracted from frozen samples by using guanidium isothiocynanate and cesium chloride gradient. DNA and RNA quality was respectively controlled on polyacrylamide gel electrophoresis and on Agilent Bioanalyzer (Agilent Technologies, Massy, France).

### Array-comparative genomic hybridization profiling and data analysis

From the 197 samples analyzed, DNA was available for 173, including 49 IBCs and 124 nIBCs. Genomic imbalances of the DNA samples were determined by using high-resolution 244K CGH microarrays (Hu-244A, Agilent Technologies, Massy, France). A pool of 13 normal male DNA was used as reference. Scanning was done with Agilent Autofocus Dynamic Scanner (G2565BA, Agilent Technologies). Data analysis was done and visualized with CGH Analytics 3.4 software (Agilent Technologies). Extraction of data (log_2_ ratio) was done from CGH analytics, while normalized and filtered log_2_ ratio were obtained from “Feature extraction” software (Agilent Technologies). Data generated by probes mapped to X and Y chromosomes were eliminated. The final dataset contained 225,388 unique probes covering 22,509 genes and intergenic regions according to the hg17/NCBI human genome mapping database (build 35). Data were analyzed using circular binary segmentation (CBS) as implemented in the DNA copy R/Bioconductor package [45] with default parameters to translate intensity measurements in regions of equal copy number, each region being defined by at least five consecutive probes. Thus, each probe was assigned a segment value referred to as its “smoothed” value.

We used two different threshold values (log_2_ ratio >|0.5|, and |1|) to distinguish low level from high level CNAs [43]. DNA copy number patterns were classified as “simplex” (very few CNAs), “complex sawtooth” (many low-level CNAs), or “complex firestorm” (low-level CNAs and recurrent amplifications) [46], [47]. Unsupervised analysis was done with the Cluster program [48] using log_2_ ratio data, complete linkage and Pearson correlation as similarity metrics. Results were displayed using TreeView [48]. To determine the robustness of the resulting tumor clusters, we used R package Pvclust [49] with multiscale bootstrap resampling using 1,000 iterations. Approximately Unbiased (AU) p-values ≥90% were used to define the significant clusters. To identify altered regions, we used the GISTIC algorithm [50], which computes for each segment through the genome a score based on the frequency of CNA combined with its amplitude, with bootstrapping to calculate the significance level (p<0.05). To identify genes with different CNA frequencies between IBCs and nIBCs, the frequency of CNAs was computed for each probe locus as the proportion of samples showing an aberration therein. Alteration frequencies were compared using the Fisher's exact test and false discovery rate (FDR) was applied to correct the multiple testing hypothesis (p<0.05) [51].

### Gene expression profiling and data analysis

Gene expression data of the 197 BCs and 4 normal breast (NB) samples, which represented 1 pool of 4 samples from 4 women, and 3 commercial pools of respectively 1, 2 and 4 normal breast RNA (Clontech, Palo Alto, CA), were quantified using whole-genome DNA microarrays (HG-U133 Plus 2.0, Affymetrix). Scanning was done with Affymetrix GeneArray scanner and quantification with Affymetrix GCOS software. Data were analyzed by the Robust Multichip Average method in R using Bioconductor and associated packages [52]. Robust Multichip Average did background adjustment, quantile normalization, and summarization of 11 oligonucleotides per gene. Before analysis, a first filtering step removed from the dataset the genes with low and poorly measured expression as defined by an expression value inferior to 100 units in all samples. All data was then log_2_-transformed for display and analysis.

Before clustering analysis, a second filtering retained the 12,813 probe sets with the most variable expression across all samples. Clustering was done with the Cluster program [48] using Pearson correlation as similarity metrics and centroid linkage clustering. Results were displayed using TreeView program [48]. The molecular subtypes of breast cancer were determined using the single sample predictor (SSP) classifier based on the list of 306 intrinsic genes as described [53]. The sample was attributed the subtype corresponding to the most correlated centroid. To develop a predictive model based on the expression of the 24 genes identified by integrated analysis (see below), we applied a logistic regression analysis using the *glm* function in R statistical package. Once defined, the model was applied to expression data to assign the “IBC-like” or the “nIBC-like” class: first for testing its robustness by leave-one-out cross-validation [54] in the learning set and by validation in an independent set of 24 IBC and nIBC samples, then for estimating its prognostic value in public nIBC datasets.

Genomic and expression data are MIAME-compliant (http://www.mged.org/Workgroups/MIAME/miame.html) and the raw data have been deposited in the MIAME-compliant GEO database (GSE23720).

### Integrated analyses of genome and expression data

Before integrated analysis, Affymetrix expression probe sets were matched with Agilent aCGH probes using the hg17/NCBI database. When multiple Affymetrix probe sets mapped to the same gene, the probe sets with an extension « _at », next « s_at », and followed by all other extensions were preferentially kept. When several probe sets with the best extension were available, the one with the highest median value was retained.

We analyzed the degree of correlation between RNA expression and CNAs for the genes and tumors (173 tumors) profiled with both platforms. For each gene with a CNA in at least two IBCs or nIBCs, a Student t-test compared the expression levels in samples with and without the corresponding CNA (FDR-corrected p<0.05). Comparative integrated analysis of IBCs and nIBCs was only applied to the genes with CNA frequencies significantly different between IBCs and nIBCs (628 genes). Genes had to satisfy three other criteria: i) frequencies of combined alterations (gain associated with overexpression *vs* other combinations, and conversely, loss associated with underexpression *vs* other combinations) different (Fisher exact test) between IBCs and nIBCs, ii) correlation (Student t-test) between CNA and expression in the 173 samples, and iii) expression different (Student t-test) between IBCs and nIBCs. In the first above-quoted step, overexpression and underexpression for a given gene were assigned using a threshold of |1| corresponding to twice the expression level found in the normal breast pool. These steps are summarized in Figure S1.

### Public gene expression data of breast cancer

To test the prognostic performance of our 24-gene signature in nIBCs, we analyzed 6 public data sets collected from five publications [55]–[59], and from the UNC Microarray Database (Table S1). When different publications included the same patients redundancy was eliminated, resulting in 1,781 different patient samples available for analysis. Before analysis, we mapped hybridization probes for the 24 genes across the two oligonucleotide-based platforms used across the series. When multiple probes were mapped to the same GeneID (EntrezGene identification number), the one with the highest variance in a particular dataset was selected to represent the GeneID. Analysis of each data set (using available normalized data) was done separately to guarantee a larger number of genes common with our signature.

### Statistical analysis

Correlations between sample groups and histoclinical factors were calculated with the Fisher's exact test for qualitative variables with discrete categories, and the Mann-Whitney test for continuous variables. Follow-up was measured from the date of diagnosis to the date of last news for patients without any event. Disease-free survival (DFS) was calculated from the date of diagnosis until the date of first relapse whatever its location (local, regional or distant) or date of death (when the relapse data was not available) using the Kaplan-Meier method. Survival was compared between groups with the log-rank test. Univariate and multivariate analyses were done using Cox regression analysis. The p-values were based on the Wald test, and patients with one or more missing data were excluded. All statistical tests were two-sided at the 5% level of significance. Analyses were done using the survival package (version 2.30), in the R software (version 2.9.1).

## Results

### Genome and transcriptome analysis of breast cancer

**Figure 1:**
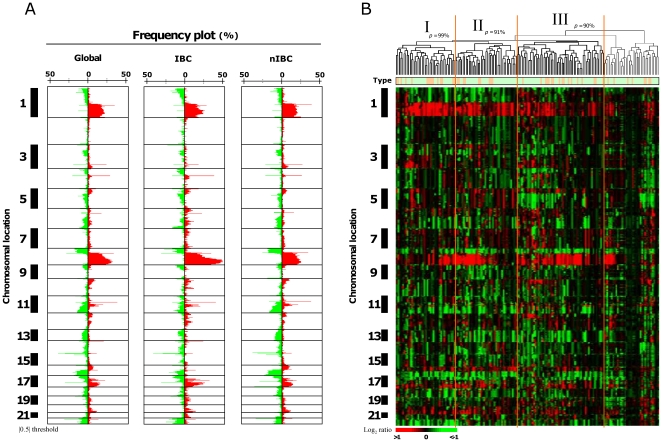
aCGH portrait of breast cancers. **A**) Frequency plots of genome CNA. Frequencies (horizontal axis, from 0 to 100%) are plotted as a function of chromosome location (from 1pter to the top, to 22qter to the bottom), for all breast cancer samples (Global, N = 173), for IBCs (N = 49), and for nIBCs (N = 124). Horizontal lines indicate chromosome boundaries. Positive and negative values indicate frequencies of tumors showing copy number increase and decrease, respectively, with gains (in red) and losses (in green). **B**) Unsupervised hierarchical clustering of genome CNAs measured for 173 breast cancers on 225,388 probes (without X and Y). Red indicates increased DNA copy number gain and green indicates decreased copy number. The bars to the left indicate chromosome locations ordered like in A). The vertical orange lines define the three significant tumor clusters (I, II and III). Above the dendrogram, *p* indicates the Approximately Unbiased (AU) p-values defined by Pvclust. Below the dendrogram, the row indicates the clinical type (green for nIBC, and orange for IBC).

We first describe the results on the whole set of tumors before addressing the specific question of IBC. High-resolution aCGH was performed on 173 samples, including 49 IBCs and 124 nIBCs. Figure 1A (left) shows the frequency of low level CNAs in all samples. As previously reported [37], [44], the three most frequently gained regions were on 1q, 8q and 17q chromosomal arms, whereas the regions frequently lost were on 8p, 11q and 16q. The median percentage of probe sets displaying a CNA in a sample was 3.5%, with a great variability between samples (range, 0.03–44%). As expected, this percentage was higher in grade 3 tumors (2.1%) than in grade 1 tumors (0.5%; p = 0.005; Mann-Whitney test).

To display the similarity of samples with respect to their genome profiles, unsupervised hierarchical clustering was applied to aCGH data of all 173 samples and all 225,388 oligonucleotide probes (excluding X and Y probes) (Figure 1B). Pvclust algorithm identified three robust tumor clusters, which correlated with the molecular subtypes of samples, and other histoclinical features (Table S2). No correlation existed with the IBC/nIBC status, suggesting similar whole-genome genomic profiles in IBCs and nIBCs, and a similar heterogeneity. Using a previous classification of genome profiles [39], [47], 55 cases (32%) were “simplex”, 39 (23%) “complex sawtooth”, and 79 (45%) “complex firestorm”. This status correlated with the grade of tumors. Only 13% of grade 3 tumors were “simplex”, whereas 87% were “complex”. Conversely, 61% of grade 1 tumors were “simplex” and 38% “complex”.

The 173 samples and 24 additional samples (197 including 63 IBCs and 134 nIBCs) were profiled using whole-genome mRNA expression microarrays. Figure S2 shows the hierarchical clustering of samples based on the expression of 12,813 probe sets. Samples were sorted into three major clusters, which strongly correlated with histoclinical features (grade, IHC data) and molecular subtypes, but not with the IBC status. As with aCGH analysis, differences due to molecular subtype greatly overcame differences due to clinical type. IBCs were scattered across the three clusters, intermixed with nIBCs, suggesting that, at the RNA level and on a whole-genome scale, they are as heterogeneous as nIBCs.

### Regions and genes with significant genome aberrations in IBCs

Our first objective was to establish the first repertoire of genome CNAs in IBC. Table S3 shows the regions with frequent low and/or high level CNAs in the 49 IBCs, defined using the GISTIC algorithm with a significance threshold of p<0.05. Sixty-five regions were gained/amplified. The top ten regions (in term of median GISTIC score) resided on 17q, 6p, 1q, 8q, 11q, 19q, and 8p chromosomal arms. Many of these regions contain genes involved in breast cancer, such as *ERBB2*, *MYC*, *CCND1* and *FGFR1*. Thirty-four regions were gained in at least 15% of IBCs. The most frequently gained region (>40%) was 8q12.1–24.3, and contained 461 genes. A total of 216 regions were lost/deleted, with the top ten (in term of median GISTIC score) distributed in 4q, 3q, 1q, 9q, 15q and 2q. Only 4 regions were lost in at least 15% of IBCs.

At the gene level, 321 genes were amplified in at least 10% of IBCs (Table S4). As expected, all were located in the 65 regions gained/amplified reported above. They included validated or potential therapeutic targets such as *ANGPT1*, *ERBB2*, *FGFR1*, *GRB7*, *MYC*, *PAK1*, *PNMT*, *PROSC*, *SQLE*, and *STK3*. Other targets such as *ADAM9* (6% of cases), *EGFR*, *FNTA*, and *IKBKB* (4%), *AKT3*, *AREG, BRAF, EREG, FYN,* and *RET* (2%) were less frequently amplified, but sometimes at a very high level (log_2_ ratio >|1|). The 15 genes that were the most frequently amplified are located in 17q12 and 8q24 (Table 2). Additionally, rare high-level amplifications targeted other potential therapeutic targets such as *DCK*, *FGF3*, *FGF10*, *FLI1*, *IL6*, *MFHAS1, ROS1* and *SRC*. Conversely, 21 genes were deleted in at least 10% of IBCs (Table S4; Table 2), and all were included in the 216 lost/deleted regions.

**Table 2:** Top 15 genes with high level CNAs in at least 10% of IBCs.

Symbol	Name	Cytoband	Amplification frequency in IBCs
*PNMT*	phenylethanolamine N-methyltransferase	17q12	29%
*PERLD1*	per1-like domain containing 1	17q12	29%
*ERBB2*	v-erb-b2 erythroblastic leukemia viral oncogene homolog 2	17q12	29%
*C17orf37*	chromosome 17 open reading frame 37	17q12	29%
*CASC3*	cancer susceptibility candidate 3	17q21.1	29%
*STARD3*	StAR-related lipid transfer (START) domain containing 3	17q12	27%
*TCAP*	titin-cap (telethonin)	17q12	27%
*GRB7*	growth factor receptor-bound protein 7	17q12	27%
*C8ORFK23*	Transcribed locus	8q24.13	24%
*C8orf54*	chromosome 8 open reading frame 54	8q24.13	24%
*PVT1*	Pvt1 oncogene homolog, MYC activator (mouse)	8q24.21	24%
*BC009730*	Homo sapiens, clone IMAGE:3884408, mRNA	8q24.21	24%
*EIF3H*	eukaryotic translation initiation factor 3, subunit H	8q24.11	24%
*EXT1*	exostoses (multiple) 1	8q24.11	24%
*SAMD12*	sterile alpha motif domain containing 12	8q24.12	24%

Symbol	Name	Cytoband	Deletion frequency in IBCs
*ADAM3A*	ADAM metallopeptidase domain 3A (cyritestin 1)	8p11.23	22%
*C14orf80*	chromosome 14 open reading frame 80	14q32.33	22%
*ADAM5P*	ADAM metallopeptidase domain 5 pseudogene	8p11.23	20%
*CRIP1*	cysteine-rich protein 1 (intestinal)	14q32.33	20%
*UGT2B17*	UDP glucuronosyltransferase 2 family, polypeptide B17	4q13.2	16%
*U06641*		4q13.2	16%
*TMPRSS11E*	transmembrane protease, serine 11E	4q13.2	16%
*THOC4*	THO complex 4	17q25.3	16%
*LOC92659*	hypothetical protein BC009233	17q25.3	16%
*NOTUM*	notum pectinacetylesterase homolog (Drosophila)	17q25.3	16%
*DEFB107A*	defensin, beta 107A	8p23.1	14%
*DEFB104A*	defensin, beta 104A	8p23.1	14%
*DEFB4*	defensin, beta 4	8p23.1	14%
*GSTT1*	glutathione S-transferase theta 1	22q11.23	14%
*RAB40C*	RAB40C, member RAS oncogene family	16p13.3	14%

### Comparative genome analysis of IBCs and nIBCs

We compared the genomic profiles of 173 samples (49 IBCs and 124 nIBCs). Globally, as shown in Figure 1A, IBCs looked like nIBCs, since both phenotypes showed similar altered regions with similar frequencies of alterations for most of them. However, some differences were visually apparent, such as the more frequent gain of 1q, 8q and 17q regions in IBCs, or the more frequent loss of 4p, 8p, 11q, and 16q regions in nIBCs. The median percentage of probe sets displaying a CNA for a sample was higher in IBCs (3.7%, range 0.01–14%) than in nIBCs (1.9%, range 0.01–26%; p = 6.1.E–253, Fisher's exact test), even if a great variability between samples existed for both types. IBCs showed a higher proportion of “complex sawtooth” and “complex firestorm” profiles than nIBCs, which conversely showed a higher proportion of “simplex” profiles (p = 0.0009, Fisher's exact test) (Figure S3).

The comparative analysis of regions with frequent low and/or high level CNA in IBCs *vs* nIBCs showed very similar results in term of number of altered regions (71 and 77 gained respectively, and 210 lost in both types) (Table S5). Many altered regions were similar. Some of them, such as 4q13.2 and 11q11, were similarly altered (similar GISTIC scores) whereas others, such as 1q21.2–q41, 8p11.21–p12, 8q11.1–q24.3, 11q13.33–q14.1, 17q11.1–q21.2, and 20q13.2–q13.33 were differentially altered (different GISTIC scores) either because of a higher frequency and/or higher amplitude of alteration. Other regions were specifically altered, such as 5p15.33 in IBCs or 3q26.1 in nIBCs.

The number of genes amplified or deleted was higher in IBCs than in nIBCs (Table S4). Only 26 genes were amplified in at least 10% of nIBCs, *vs* 321 in IBCs. As expected, these genes are located within amplicons classically described in breast cancer on 8p, 11q and 17q. Sixteen genes (*vs* 21 in IBCs) were deleted in at least 10% of nIBCs. Supervised analysis identified 9,148 probes, representing 628 unique sequences/genes, with different CNA frequency in IBCs and nIBCs (Tables S6A–B). A total of 514 genes displayed gains and/or amplifications differentially associated with IBCs *vs* nIBCs, including 484 genes gained/amplified more frequently in IBCs, and only 30 gained more frequently in nIBCs (Table S6). Compared to nIBCs, IBCs were associated with the gain of 382 genes scattered through 13 chromosomal arms (1p, 4q, 5p, 6p, 6q, 7p, 8q, 10q, 12q, 14q, 17q, 19p, and 20q). Figure S4 shows an example of genomic profiles for the 6q21 region. The frequently gained 8q21.2–24.3 region included more than 230 gained genes, including *MTDH* (8q22.1), *RRM2B*, *AZIN1*, *FZD6* (8q22.3), *ANGPT1*, *EIF3E* (8q23.1), *DCC1*, *MTBP* (8q24.12) and *ATAD2*, *SQLE* (8q24.13). By contrast, only 30 genes, scattered through two chromosomal arms (3q and 16p), were more specifically gained in nIBCs, including *MYH11*, *C16orf63* (16p13.11), *ERCC4* (16p13.12), and *A2BP1* (16p13.3). Thus, specific gains were more frequently observed in IBCs than in nIBCs. The mean frequency of tumors displaying a gain for one gene of these respective lists was 31% for IBCs (maximum 53% for genes located in the 8q22.3–24.11 region), and only 9% for nIBCs (maximum 10% for genes located in the 16p13.11 region). A total of 189 genes were more frequently amplified in IBCs than in nIBCs. Most of them were located within five chromosomal arms (1q, 8p, 8q, 17q, and 18p). Several genes such as *RAD21* (8q24.11), *MTBP* (8q24.12), *MYC*, *PVT1* (8q24.21), *ERBB2* (17q12), and *CASC3* (17q21.1) are known cancer-related genes. Ninety-nine of these 189 genes were amplified in at least 10% of IBCs. The mean frequency of IBCs with amplification for one of these genes was 14% (maximum 29% for genes such as *ERBB2* located in the 17q12 region). In contrast, no gene was more frequently amplified in nIBCs than in IBCs.

A total of 114 genes displayed genomic losses and/or deletions differentially associated with IBCs *vs* nIBCs, including 68 genes lost/deleted more frequently in IBCs, and 46 more frequently in nIBCs (Table S7). Fifty-nine genes were more specifically lost in IBCs, affecting 7 chromosomal arms (4p, 5q, 6q, 12p, 15q, 19q, and 22q). Examples include *EMB* (5q11.1), *RPS5*, *UBE2M* (19q13.43), *SLIT2* (4p15.31) and *EZR* (6q25.3). Conversely, 46 genes were more specifically lost in nIBCs, affecting two chromosomal arms (8p, and 16q). Examples include *PKD1L2* (16q23.2), *FOXC2* and *FOXF1* (16q24.1). Loss and amplification of *BC028701*, *FKSG2* and *KCNU1* (8p12), were correlated with nIBCs and IBCs, respectively. The mean frequency of tumors displaying a loss for one gene of these respective lists was 11% for IBCs (maximum 24% for genes located in the 6q27 and 22q13.1 regions), and 20% for nIBCs (maximum 21% for genes located in the 16q24.1 region). In IBCs homozygous deletions (no copies assuming a modal diploid genome) targeted 9 genes located on five chromosomal arms (3q, 8p, 13q, 14q, and 18q). Four of them were deleted in at least 10% of IBCs. The mean frequency of IBCs with deletion for one of these genes was 10% (maximum 14% for genes located in the 8p23.1 region). No homozygous deletion was more frequently found in nIBCs.

Thus, among the 628 genes (514 gained/amplified and 114 lost/deleted) with CNAs differentially represented between IBCs and nIBCs, 552 (484 and 68; 88%) were associated with IBCs and 76 (30 and 46; 12%) with nIBCs.

### Correlations between gene expression and CNA

We compared the degree of CNA-driven mRNA up and downregulation in 49 IBCs and 124 nIBCs profiled on both platforms by analyzing the 13,127 genes common to the two platforms and retained after filtering based on the expression levels.

In IBCs, from all genes with a CNA (gains/amplifications and losses/deletions) in at least two samples, 10.4% of gained genes exhibited mRNA expression correlated with the gain, and 1.5% of lost genes exhibited expression correlated with the loss (Table S8). In nIBCs, these respective features were 10.4% and 9.5%. For the gained genes, the percentages of correlations were similar in IBCs and nIBCS but for the lost genes, this percentage was 7-fold more important in nIBCs than in IBCs (p<1.E-12, Fisher's exact test; OR = 0.14 (CI95% 0.09–0.20)).

### Integrated comparative analysis and IBC-specific candidate genes

To identify potential IBC-specific candidate genes, we compared in the 173 IBCs and nIBCs only the genes with deregulated mRNA expression in relation to CNA (Figure S1).

**Figure 2:**
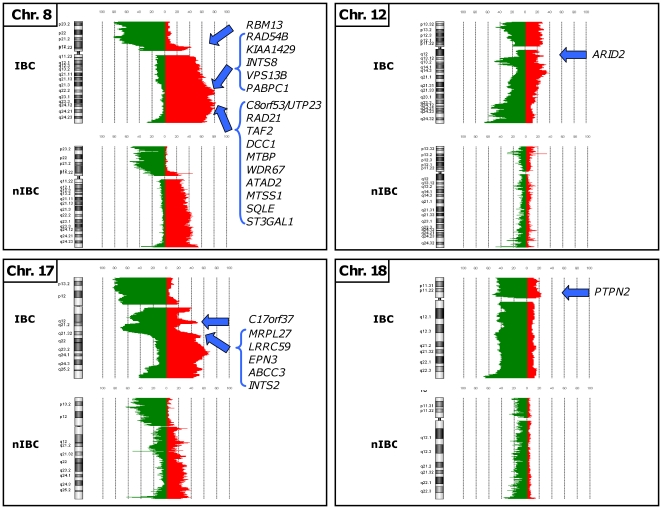
Chromosomal location of the IBC candidate oncogenes. The 24 candidate oncogenes defined by comparative integrated analysis are shown associated with their corresponding chromosome CNA frequency plot in 49 IBCs and 124 nIBCs. A threshold value of log_2_ ratio >|0.5| was used to draw chromosome CNA frequency plots using CGH Analytics® software.

Out of the 628 genes identified by supervised analysis with CNA frequencies different between IBCs and nIBCs, 500 were present on the Affymetrix microarrays. They were represented by 748 probe sets on these microarrays, and 4,259 probes on the Agilent microarrays. From these 500 genes, we identified 24 genes whose expression was deregulated in relation to CNA with significant differences between IBCs and nIBCs (Table 3; Table S9; Figure 2). In all cases, the transcriptional deregulation was associated with IBCs only and corresponded to an overexpression related to a gain (21 genes) and/or amplification (13 genes). By definition, these 24 genes were also overexpressed in IBCs as compared to nIBCs. Twenty of these genes are located in 8q22–q24 and 17q21, including *PAPBC1*, *RAD21*, *ATAD2, MTSS1*, *SQLE*, *ST3GAL1*, *C17orf37*, *ABCC3* and *PTPN2*, previously reported as cancer-related genes. In contrast, no candidate gene was both downregulated and loss-targeted in IBCs.

**Table 3:** List of 24 candidate genes with gain or amplification correlated with overexpression showing significant frequency differences between IBCs and nIBCs.

Symbol	Name	Cytoband
*RBM13*	RNA binding motif protein 13	8p12
*RAD54B*	RAD54 homolog B (S. cerevisiae)	8q22.1
*KIAA1429*	KIAA1429	8q22.1
*INTS8*	integrator complex subunit 8	8q22.1
*VPS13B*	vacuolar protein sorting 13 homolog B (yeast)	8q22.2
*PABPC1*	poly(A) binding protein, cytoplasmic 1	8q22.3
*C8orf53/UTP23*	chromosome 8 open reading frame 53	8q24.11
*RAD21*	RAD21 homolog (S. pombe)	8q24.11
*TAF2*	TAF2 RNA polymerase II, TATA box binding protein (TBP)-associated factor, 150 kDa	8q24.12
*DCC1*	defective in sister chromatid cohesion homolog 1 (S. cerevisiae)	8q24.12
*MTBP*	Mdm2, transformed 3T3 cell double minute 2, p53 binding protein (mouse) binding protein, 104kDa	8q24.12
*WDR67*	WD repeat domain 67	8q24.13
*ATAD2*	ATPase family, AAA domain containing 2	8q24.13
*MTSS1*	CDNA FLJ12372 fis, clone MAMMA1002446	8q24.13
*SQLE*	squalene epoxidase	8q24.13
*ST3GAL1*	ST3 beta-galactoside alpha-2,3-sialyltransferase 1	8q24.22
*ARID2*	AT rich interactive domain 2 (ARID, RFX-like)	12q12
*C17orf37*	chromosome 17 open reading frame 37	17q12
*MRPL27*	mitochondrial ribosomal protein L27	17q21.33
*LRRC59*	leucine rich repeat containing 59	17q21.33
*EPN3*	epsin 3	17q21.33
*ABCC3*	ATP-binding cassette, sub-family C (CFTR/MRP), member 3	17q21.33
*INTS2*	integrator complex subunit 2	17q23.2
*PTPN2*	protein tyrosine phosphatase, non-receptor type 2	18p11.21

**Figure 3:**
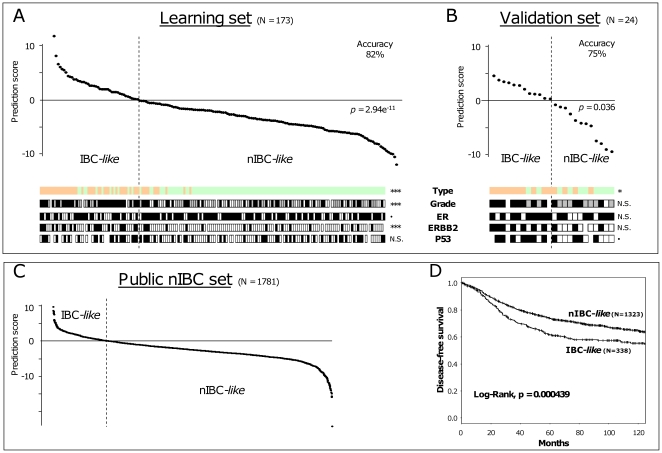
Discriminative power of the 24-gene signature and prognostic value in nIBCs. **A**) Classification of the 173 breast cancers (124 nIBCs and 49 IBCs) from which we have generated the 24-gene IBC signature (learning set) by leave-one-out cross-validation. Samples are ordered from left to right according to the decreasing prediction score defined by the 24-gene model. The vertical dashed line indicates the threshold 0 that separates the “IBC-like class” (left of the line) and the “nIBC-like class” (right to the line). Below the curve are some histoclinical and molecular features of the samples: from top to bottom, clinical type (green for nIBC, and orange for IBC), SBR grade (white for grade I, grey for II, and black for III), IHC ER, ERBB2, and P53 status (white for negative, and black for positive). The p-value of correlations between the two tumor classes (“IBC-like” and “nIBC-like) and these features is indicated as follows: ***, <0.001; **, <0.01; *, <0.05; •, <0.1; NS, not significant. **B**) The classification is validated in the set of 24 independent samples (10 nIBCs and 14 IBCs). **C**) The same classification method is applied to 1,781 publicly available nIBCs, allowing defining two classes: “IBC-like” and “nIBC-like”. **D**) Kaplan-Meier DFS curves of the two nIBC classes defined in C.

To validate the association of these 24 genes with the IBC/nIBC distinction, we analyzed the 24 remaining breast tumors (14 IBCs and 10 nIBCs) only profiled on our Affymetrix platform. None of them had been included in the 173 tumors from which the 24 gene-list had been derived. First, we defined an IBC/nIBC genomic classifier by applying logistic regression to the expression data of the 24 genes in the 173 samples (learning set). As expected, the rate of accurate resulting classification was high (86%). Leave-one-out cross-validation gave similar results with a rate of 82% (p = 2.94E-11, Fisher's exact test; Figure 3A). Then, we applied the predictor to our validation set of 24 samples (Figure 3B): 75% of samples (10/14 IBCs and 8/10 nIBCs; p = 0.03, Fisher's exact test) were correctly classified, suggesting the robustness of the classifier.

Because of the differences between IBC and nIBC regarding some histoclinical features such as grade, ER, ERBB2 and P53 status, we verified that our 24-gene signature was more associated to the IBC/nIBC distinction than to any of these features. This was done by comparing the p-values testing the correlations between the “IBC-like”/“nIBC-like” classes and each feature (Figure 3A–B). This was further confirmed by regression analysis on the whole set of samples testing and comparing the ability of each variable (24-gene signature, grade, ER, ERBB2 and P53 status) to discriminate IBC from nIBC (Table 4). Three features were significant in univariate analysis (24-gene signature, ERBB2 and P53 status), and two, including the 24-gene signature, remained significant in multivariate analysis. Altogether, these data indicated that our 24-gene signature is more linked to the distinction IBC/niBC than to other potential confounding variables.

**Table 4:** Uni- and multivariate logistic regression analyses of IBC/nIBC distinction, including the 24-gene signature.

	Univariate			Multivariate		
	N	coef	*p* **	N	coef	*p* **
ER pos *vs* neg	197	−0.5	0.11			
ERBB2 pos *vs* neg	185	2.06	3.53E-08	153	2.44	5.34E-05
P53 pos *vs* neg	163	1.28	6.08E-04	153	0.42	0.45
Grade 2–3 *vs* 1	190	18.02	0.99			
nIBC-like *vs* IBC-like	197	−3.23	2.29E-15	153	−3.05	3.12E-08

*Logistic regression analysis using the *glm* function in *R*'s statistical package.

**Significance was estimated by specifying a binomial family for model with a logit link.

### Prognostic value of the 24-gene signature

Given the poor prognosis of IBCs, we hypothesized that the 24-gene predictor, if biologically relevant with respect to the IBC/nIBC distinction, might be prognostic in breast cancer. We thus tested its prognostic value in a series of 1,781 clinically annotated nIBCs. Using our 24-gene model (the number of genes common with each data set ranged from 17 to 24), we attributed to each sample an “IBC-like” or “nIBC-like” profile. All series were then pooled, resulting in 338 nIBCs with an “IBC-like” profile and 1,323 with a “nIBC-like” profile (Figure 3C).

We compared the DFS of these two nIBC classes for the 1,420 patients with available clinical outcome. With a median follow-up of 91 months after diagnosis (range, 1 to 220), the “nIBC-like” class had a 5-year DFS of 73%, better than the survival of the “IBC-like” class with a 5-year DFS of 61% (p = 4.4.E-4, log-rank test). Survival curves are shown in Figure 3D.

We then performed univariate and multivariate DFS analyses (Table 5). In addition to the “IBC-like” or “nIBC-like” profile, we tested the variables most frequently annotated in the six data sets: patients' age, pathological tumor size, axillary lymph node status, and grade, and IHC ER and PR status (ERBB2 status not available). All features, except age and PR status, were significant in univariate analysis. The hazard ratio (HR) for relapse was 1.42 for “IBC-like” tumors compared to “nIBC-like” tumors ([95%CI 1.17–1.73], p = 4.7.E-4). In multivariate analysis, these five variables, including the 24-gene model-based classification, retained significant prognostic value, suggesting that our IBC signature is an independent prognostic feature in nIBC, with a HR for relapse for “IBC-like” tumors *vs* “nIBC-like” tumors equal to 1.34 ([95%CI 1.06–1.70], p = 0.015). This observation confirmed that the 24-gene signature contained some prognostic information, which might explain the worse prognosis of IBCs.

**Table 5:** Cox univariate and multivariate analyses of DFS in the public series of nIBCs.

	Univariate Analysis			Multivariate analysis		
	N	HR [95%CI]	p	N	HR [95%CI]	p
Age,	1372	0.917 [0.76–1.11]	0.37			
>50y *vs* ≤50y						
Pathological axillary lymph node status,	1627	1.33 [1.12–1.58]	0.0013	1235	1.25 [1.01–1.54]	3.90E-02
pos *vs* neg						
Pathological tumor size,	1352	2.08 [1.69–2.56]	3.00E-12	1235	1.71 [1.37–2.15]	3.00E-06
pT2-4 *vs* pT1						
Grade,	1291	2.57 [1.87–3.54]	7.80E-09	1235	1.88 [1.33–2.65]	3.60E-04
2–3 *vs* 1						
IHC ER status,	1638	0.571 [0.47–0.69]	3.40E-09	1235	0.56 [0.45–0.7]	3.00E-07
pos *vs* neg						
IHC PR status,	453	0.73 [0.49–1.09]	0.12			
pos *vs* neg						
IBC-like *vs* nIBC-like	1661	1.42 [1.17–1.73]	0.00047	1235	1.34 [1.06–1.7]	1.50E-02

## Discussion

We applied high-throughput molecular analyses to a large series of IBCs and nIBCs. Because copy-number changes drive a considerable proportion of the transcriptional changes [34], we compared genome copy number and expression profiles to identify potential IBC-specific candidate genes. To our knowledge, this is the first high-resolution aCGH study of IBC, the first integrated genomic analysis for IBC vs nIBC comparison, and the largest series of IBCs profiled using high-resolution genomic analytic tools.

### Genomic alterations in IBC

Because of the high number of CNAs observed in breast cancer, we used stringent log_2_ ratio threshold values to define the most specific genomic aberrations. We did not separate IBCs from nIBCs on the basis of whole genome profiles, which were globally very close, suggesting that IBCs are as heterogeneous as nIBCs at the genome level and that different obvious genome alterations are not what distinguish them. However, the number and frequency of CNAs were more important in IBCs than in nIBCs, as well as the proportion of “complex sawtooth” and “complex firestorm” profiles, clearly suggesting that the genomic differences between them are not due to a possible damping of the nIBC signal by higher contamination with normal tissue. Among the 628 genes with CNAs differentially represented between IBCs and nIBCs, 88% were associated with IBCs and only 12% with nIBCs. These results indicate a higher degree of genomic instability in IBCs, in agreement with their high grade, frequency of P53 mutations and their aggressiveness. Given this genomic complexity and heterogeneity of IBCs, and the low degree of differences observed with nIBCs globally, future studies should ideally compare IBCs and nIBCs within molecular subgroups defined by expression (molecular subtypes) and/or by CNA (simplex, complex sawtooth, complex firestorm) profiles as recently suggested [60]. In this context, and given the scarcity of IBC, international collaborations are underway for collecting enough IBC samples that should allow identifying genomic alterations perhaps more specific of IBC.

However, even if the number of profiled samples is relatively small, our series represents the largest tumor set reported in literature, and its study provides several interesting results. We identified 65 regions with gain/amplification and 216 with loss/deletion in IBCs. Few data are available in literature regarding the genomic imbalances in IBCs [40]–[42], and none has been done using high-resolution aCGH. A study of loss of heterozygosity at 71 microsatellite markers in 66 IBCs [41] reported deletions of various chromosomal regions including 3p14–p21.2, 6p, 8p22, 11q22–q23, 11q24–q25, 13q14, and especially 17q21, more frequently than in nIBCs. Our results show some overlap with these results (Table S3). We here identified four 17q21 genes, *NSF*, *ARL17P1*, *ARL17* and *KIAA1267* targeted by loss in 17% of IBCs (Table S3).

In both IBCs and nIBCs the number of regions with CNAs was very close and many altered regions were similar, suggesting that common genes are involved in both types. However, some common regions displayed higher frequency and/or higher amplitude of alteration in IBCs, and other regions were specifically altered in IBCs such as 5p15.33, previously associated with poor prognosis in breast [61] and bladder cancers [62].

At the gene level, the results were similar. Many genes were similarly altered in IBCs and nIBCs in terms of frequency and amplitude of aberrations. However, a larger number of genes displayed recurrent amplifications in IBCs than in nIBCs. Some genes, such as *CCND1* and *FGFR1*, were amplified at the same frequency in the two types, whereas others (99 out of 321 recurrently amplified, 31%), such as *ERBB2* and *MYC*, were more often amplified in IBCs. Of note, all high-level CNAs differentially represented between the two types were associated with IBCs, and none with nIBCs. Several of them code for validated or potential therapeutic targets, which could contribute to enlarge our therapeutic armament in IBC. The difference between IBCs and nIBCs was much less important regarding the genes recurrently deleted. The deletion of the *RB1* tumor suppressor gene likely contributes to the genomic instability in IBC.

### IBC and transcriptional deregulation

Whole-genome gene expression profiling did not distinguish IBCs and nIBCs more than did aCGH. To our knowledge, the information about the relationship between gene CNAs and mRNA expression in IBC is scarce in the literature. In the present study, we restricted the analysis of expression data to genes with DNA CNA. Having identified the genes with a CNA in at least two IBCs and in at least two nIBCs, we determined those whose mRNA expression correlated with the CNA. In both tumor types, ∼10% of gained genes presented such correlation, in agreement with previous observations obtained with low resolution techniques and less samples in nIBC series [34], [63]. In contrast, this percentage for the genes with loss/deletion was smaller in IBCs (1.4%) than in nIBCs (9.5%), suggesting that epigenetic mechanisms might be more operational in IBCs than in nIBCs.

### IBC candidate genes

Integrated analysis of aCGH and expression data identified 24 genes as potential IBC-specific candidate oncogenes, whereas no IBC-specific gene inactivated by loss was found. This does not rule out the likely existence of IBC-specific tumor suppressor genes inactivated by other mechanisms, as well as the existence of a gene expression signatures identified by the sole comparison of whole-genome expression data of IBC vs nIBC. Importantly, the discriminative power of the predictive model - built from the expression levels of these 24 genes - was validated in an independent sample set. Furthermore, this model was an independent prognostic feature in a multicentric series of 1781 nIBCs, indirectly validating its association with IBC, known to be more aggressive than nIBC.

Whether these genes are causative or even predictive of the IBC phenotype in a biological sense or reflect aggressiveness or another associated phenomenon remain to be explored by further in-depth experimental analyses. Several encode proteins involved in the protein translation and transport: MRPL27, a component of mitochondrial ribosomes; VPS13B, involved in vesicle-mediated sorting and transport of proteins within the cell; ABCC3, an ATP-binding cassette transporter, and PABPC1 [64]. Increasing evidence points to a crucial role of translational regulation in cancer development and progression, notably in IBC [65]. PABPC1 is a poly(A)-binding protein (PABP) required for translation initiation. Its interaction with the translation initiation factor eIF4G is crucial for the translational stimulatory effect conferred by the poly(A) tail. In IBC, eIF4G1 reprograms the protein synthetic machinery for specifically increasing the translation of certain mRNAs, notably that encoding p120 catenin, resulting in an increased stabilization of E-cadherin, and that encoding VEGF [66, Silvera, 2009 #2199]]. E-cadherin stabilization maintains the structure of tumor emboli, allowing them to survive and to metastasize as entire structures. VEGF expression accounts for high levels of angiogenesis in IBC and resistance to hypoxia. Our result suggests that PABPC1 could also participate and potentiates this process, allowing IBC cells to adapt to the persistent hypoxia they experience as tumor emboli. Other genes are associated with cell cycle progression: *RBM13*/*MAK16* [67], *TAF2*, *ATAD2*, *UTP23*, *MTBP*, and *DSCC1*. TAF2 is a general transcription factor particularly involved in the G2/M transition [68]. ATAD2, as target of E2F, ER and coactivator of MYC [69], links the 3 corresponding pathways, and likely contributes to the aggressiveness of disease through the enhancement of MYC-dependent transcription [70]. UTP23 [71] is a component of the small subunit processome, required for ribosome biogenesis and cell cycle progression at G1. MTBP regulates the E3 ubiquitin ligase activity of MDM2, a critical negative regulator of p53 function [72]. DSCC1 is a component of an alternative replication factor C complex that loads PCNA onto DNA during S phase. Genes of the signature encode proteins involved in RNA processing and transcription: the TAF2 and ATAD2 transcriptional regulators, ARID2, which facilitates ligand-dependent transcriptional activation by nuclear receptors [73], UTP23; INTS2 and INTS8, subunits of the integrator complex, which associates with RNA polymerase II and mediates 3-prime end processing of snRNAs (Baillat, D, Cell 2005). Other genes are associated with metabolism. *ST3GAL1* encodes a glycosyltransferase that induces aberrant glycosylation of MUC1 in breast cancer [4], [74]. *SQLE* encodes a key enzyme of cholesterol biosynthesis, whose expression is associated with poor survival in nIBCs [75]. Two genes, *RAD21* and *RAD54B*, are associated with DNA repair [76]. Finally, four genes (*PTPN2*, *MTSS1*, *EPN3*, and *C17ORF37*) are associated with cell migration and adhesion and/or poor prognosis of breast cancer. The PTPN2**/**TC-PTP phosphatase stimulates the ERK pathway [77], and its decreased expression markedly impairs IGF2 induced MCF7 migration [78]. The presence of *MTSS1*, which encodes for metastasis suppressor 1 [79] appears paradoxal, given its favorable prognostic impact in breast cancer [80]. However, it has been suggested that MTSS1 is unlikely to be a metastasis suppressor, but interacted with RAC, actin and actin-associated proteins to modulate lamellipodia formation [81]. Epsin 3 (EPN3) is involved in extracellular matrix-epithelial cells interactions [82]. C17orf37, whose expression correlates with grade and stage of nIBC [83], promotes invasion and migration of prostate cancer cells by enhancing secretion of uPA, MMP9 and VEGF through NF-_k_B pathway [84]. Altogether, these different processes (protein translation and transport, cell cycle, RNA processing and transcription, metabolism, cell migration) are consistent with the hyperproliferative and invasive phenotype of IBC. Some of them (protein processing, RNA translation, proliferation and lipid metabolism) have been previously reported as overrepresented among genes or pathways associated with IBC [29], [31].

In conclusion, we report the first description of genomic profiles of IBCs, on a large sample size and with a high-resolution aCGH platform, and the first integrated genomic analysis comparing IBC vs nIBC. This repertoire of whole-genome CNAs in IBCs may serve of basis for further investigations. We show the genomic complexity and heterogeneity of IBCs, which globally look like nIBCs. Many genes targeted by CNA - some of them specific of IBC - have not been previously reported in breast cancer. We have identified 24 IBC-specific potential oncogenes that could explain, at least partially, the IBC phenotype and its aggressiveness, and lead to the development of new therapeutic strategies. As such, they represent new candidates for further clinical and functional validation in IBC. One of them, *PABPC1*, is particularly interesting as it likely potentiates the role of an alteration recently discovered as essential in IBC pathogenesis. Our findings, as well as the comprehensive database of CNA and mRNA expression generated, constitute a novel step towards the goal of better understanding, and perhaps treating, IBC, even if other alterations of the tumor, as well as those of its microenvironment, at other molecular levels such as DNA mutations, epigenetic regulations, microRNAs [85], proteins and others [4], [25] still need to be identified.

## Supporting Information

Figure S1 Integrated comparative analysis of IBcs and nIBCs with the three successive steps numbered 1, 2 and 3).
(PPT)

Figure S2 Whole-genome expression profiling of IBCs and nIBCs. **A**) Hierarchical clustering of 197 samples and 12,813 probe sets with significant variation in mRNA expression level across the samples. Each row of the data matrix represents a gene and each column represents a sample. Expression levels are depicted according to the color scale shown at the bottom. Red and green indicate expression levels respectively above and below the median. The magnitude of deviation from the median is represented by the color saturation. The dendrogram of samples (above matrixes) represents overall similarities in gene expression profiles and is zoomed in B. Colored bars to the right indicate the locations of 6 gene clusters of interest (ECM means extra-cellular matrix). **B**) Dendrograms of samples. *Top*, three large groups of tumor samples (designated I to III) are evidenced and delimited by orange vertical lines. Below the dendrogram, are some histoclinical and molecular features of the samples: from top to bottom, clinical type (green for nIBC, and orange for IBC), SBR grade (white for grade I, grey for II, and black for III), IHC ER, ERBB2, and P53 status (white for negative, and black for positive), and intrinsic molecular subtypes (dark blue for luminal A, light blue for luminal B, red for basal, pink for ERBB2-overexpressing, and green for normal-like).
(PPT)

Figure S3 Proportion of genomic patterns in IBCs and nIBCs.
(PPT)

Figure S4 6q21 gains are more frequent in IBCs than nIBCs. Profiles of chromosome 6 show higher 6q21 gain frequency in IBC tumors than in nIBC (**A**). Regional genomic profiles were established with CGH analytics® software (Agilent Technologies), for IBC and nIBC cases (panels **B** and **C**) both within the genomic interval [105.9–114.7 Mb] of the long arm of the chromosome 6 (hg17 human genome mapping; build 35 from NCBI). Profiles are distinguishable by different colors corresponding to different cases. Several IBC cases showed 6q21 gain or regional or focused amplification (**B**), whereas only two nIBC cases displayed a regional amplification (**C**).
(PPT)

Table S1 Description of the public nIBC data sets.
(XLS)

Table S2 Histoclinical correlations of the three aCGH-clustered groups.
(XLS)

Table S3 Regions with CNAs in IBCs.
(XLS)

Table S4 Genes with high level CNAs in IBCs and/or nIBCs.
(XLS)

Table S5 Regions with CNAs in nIBCs.
(XLS)

Table S6 Genes with copy number gain/amplification frequencies significantly different between IBCs and nIBCs.
(XLS)

Table S7 Genes with copy number loss/deletion frequencies significantly different between IBCs and nIBCs.
(XLS)

Table S8 Correlation between DNA CNAs and RNA expression for the 13,127 genes present on both platforms, in IBCs, and in nIBCs.
(XLS)

Table S9 List of 24 genes with gain or amplification correlated with overexpression showing significant frequency differences between IBCs and nIBCs.
(XLS)
