# Supplementary material for: High-Resolution Comparative Genomic Hybridization of Inflammatory Breast Cancer and Identification of Candidate Genes
Source: PLoS One. 2011 Feb 9;6(2):e16950. doi: 10.1371/journal.pone.0016950 (PMC3037286; doi:10.1371/journal.pone.0016950)
Supplement: Figure S1 — Integrated comparative analysis of IBcs and nIBCs with the three successive steps numbered 1, 2 and 3). (PPT) [file pone.0016950.s001.ppt]

## Slide 1
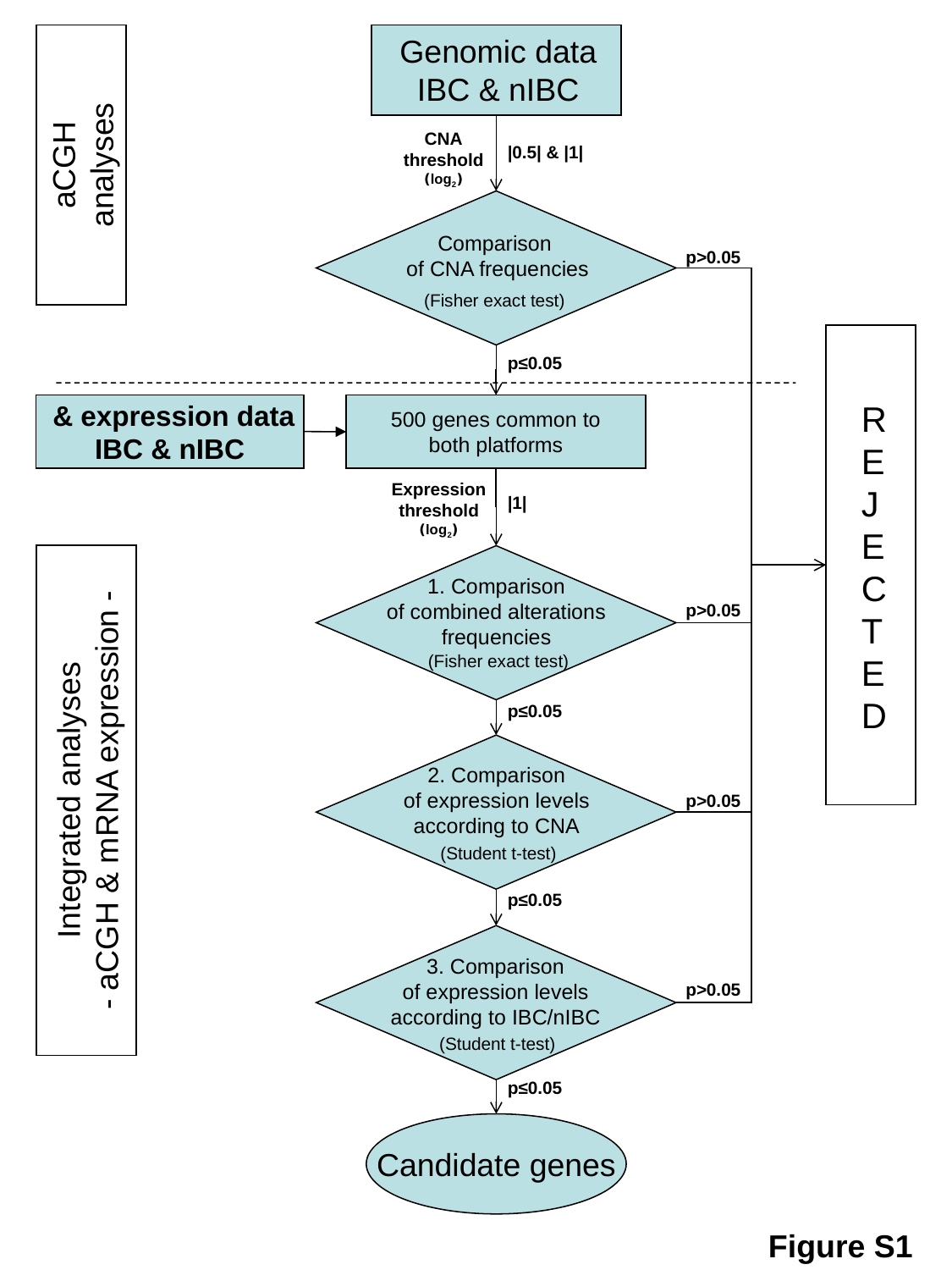

Genomic data
IBC & nIBC
aCGH
analyses
CNA
threshold
(log2)
|0.5| & |1|
Comparison
of CNA frequencies
p>0.05
(Fisher exact test)
p≤0.05
REJECTED
 & expression data
IBC & nIBC
500 genes common to both platforms
Expression
threshold
(log2)
|1|
1. Comparison
of combined alterations frequencies
p>0.05
(Fisher exact test)
p≤0.05
Integrated analyses
- aCGH & mRNA expression -
2. Comparison
of expression levels according to CNA
p>0.05
(Student t-test)
p≤0.05
3. Comparison
of expression levels according to IBC/nIBC
p>0.05
(Student t-test)
p≤0.05
Candidate genes
Figure S1
