# Supplementary material for: High-Resolution Comparative Genomic Hybridization of Inflammatory Breast Cancer and Identification of Candidate Genes
Source: PLoS One. 2011 Feb 9;6(2):e16950. doi: 10.1371/journal.pone.0016950 (PMC3037286; doi:10.1371/journal.pone.0016950)
Supplement: Figure S2 — Whole-genome expression profiling of IBCs and nIBCs. A) Hierarchical clustering of 197 samples and 12,813 probe sets with significant variation in mRNA expression level across the samples. Each row of the data matrix represents a gene and each column represents a sample. Expression levels are depicted according to the color scale shown at the bottom. Red and green indicate expression levels respectively above and below the median. The magnitude of deviation from the median is represented by the color saturation. The dendrogram of samples (above matrixes) represents overall similarities in gene expression profiles and is zoomed in B. Colored bars to the right indicate the locations of 6 gene clusters of interest (ECM means extra-cellular matrix). B) Dendrograms of samples. Top, three large groups of tumor samples (designated I to III) are evidenced and delimited by orange vertical lines. Below the dendrogram, are some histoclinical and molecular features of the samples: from top to bottom, clinical type (green for nIBC, and orange for IBC), SBR grade (white for grade I, grey for II, and black for III), IHC ER, ERBB2, and P53 status (white for negative, and black for positive), and intrinsic molecular subtypes (dark blue for luminal A, light blue for luminal B, red for basal, pink for ERBB2-overexpressing, and green for normal-like). (PPT) [file pone.0016950.s002.ppt]

## Slide 1
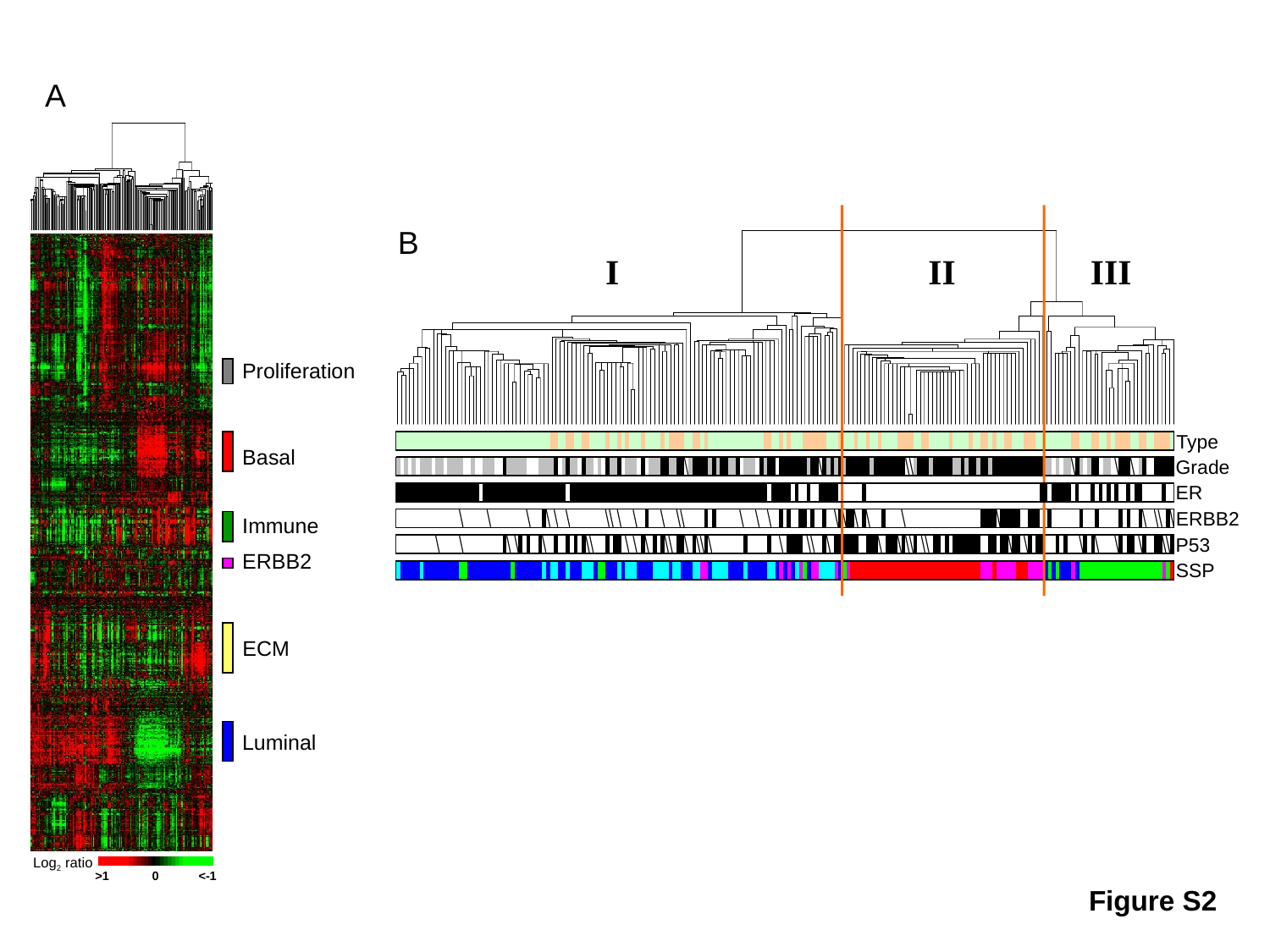

A
B
I
II
III
Proliferation
Type
Basal
Grade
ER
ERBB2
Immune
P53
ERBB2
SSP
ECM
Luminal
Log2 ratio
>1
0
<-1
Figure S2
