# Supplementary material for: High-Resolution Comparative Genomic Hybridization of Inflammatory Breast Cancer and Identification of Candidate Genes
Source: PLoS One. 2011 Feb 9;6(2):e16950. doi: 10.1371/journal.pone.0016950 (PMC3037286; doi:10.1371/journal.pone.0016950)
Supplement: Figure S3 — Proportion of genomic patterns in IBCs and nIBCs. (PPT) [file pone.0016950.s003.ppt]

## Slide 1
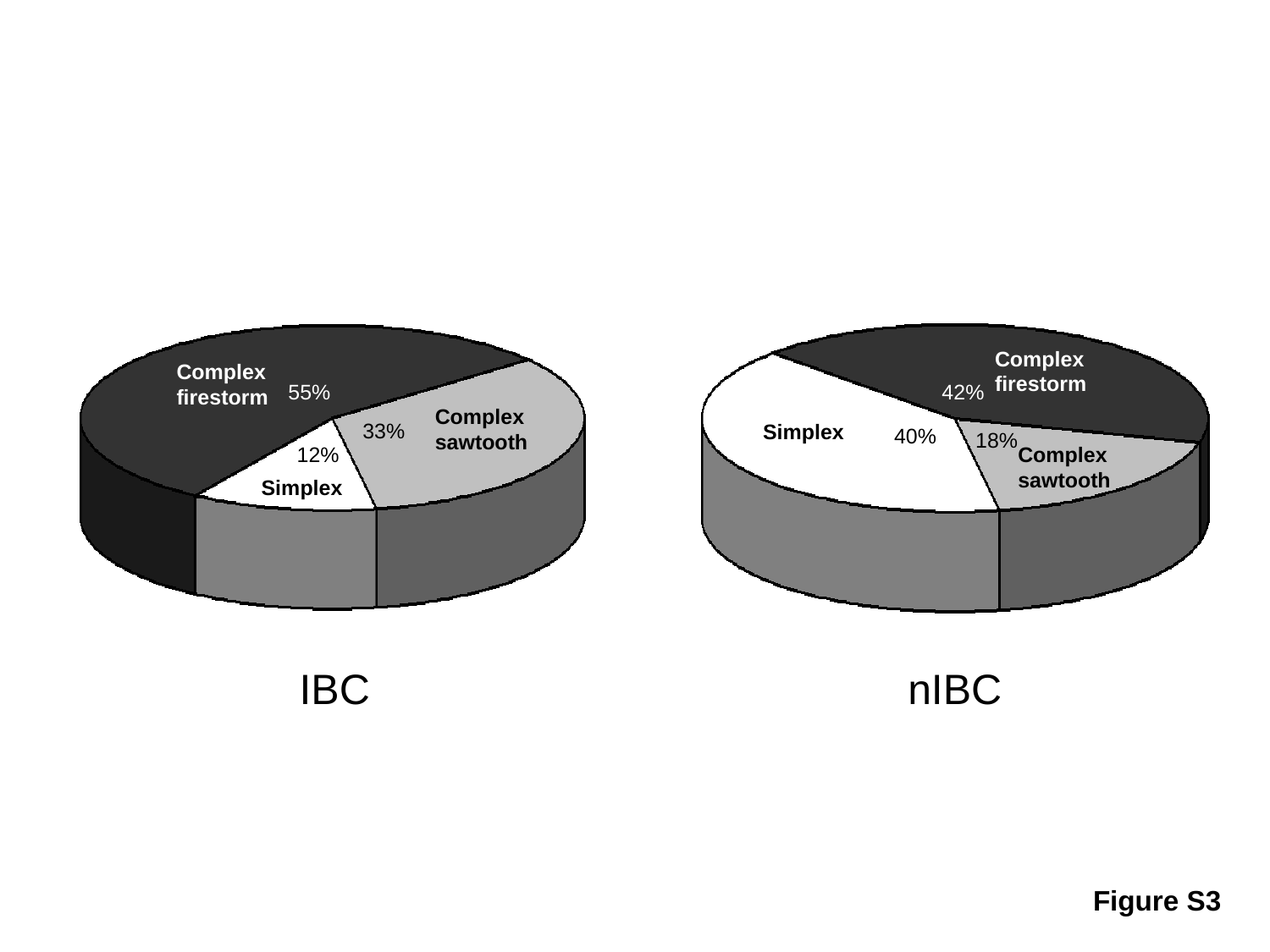

Complex
firestorm
Complex
firestorm
42%
55%
Complex
sawtooth
40%
33%
Simplex
18%
12%
Complex
sawtooth
Simplex
IBC
nIBC
Figure S3
