# Supplementary figures and images for: High-Resolution Comparative Genomic Hybridization of Inflammatory Breast Cancer and Identification of Candidate Genes
Source: PLoS One. 2011 Feb 9;6(2):e16950. doi: 10.1371/journal.pone.0016950 (PMC3037286; doi:10.1371/journal.pone.0016950)

## Slide 1
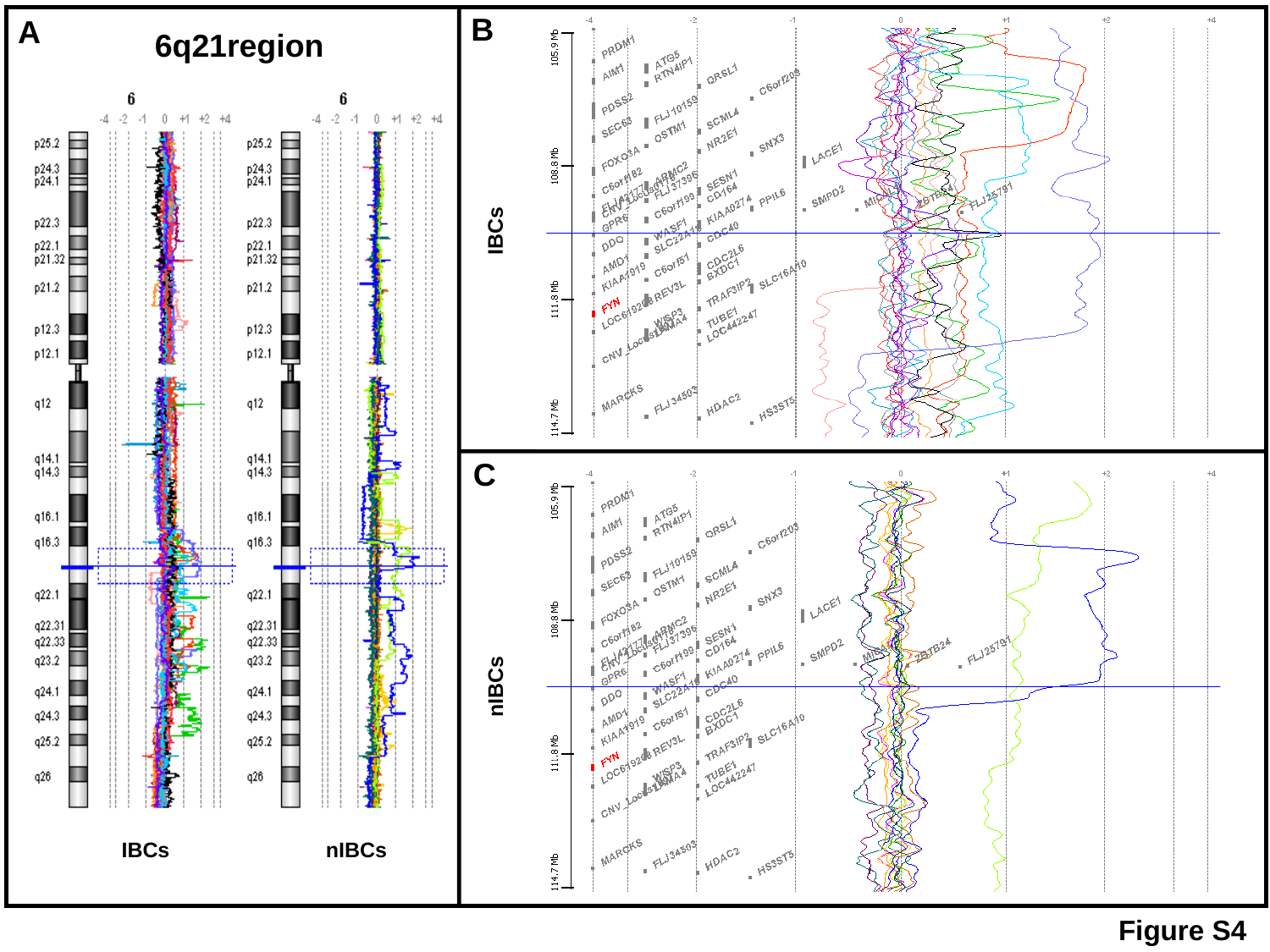

B
A
6q21region
IBCs
C
nIBCs
IBCs
nIBCs
Figure S4

Supplement: Figure S4 — 6q21 gains are more frequent in IBCs than nIBCs. Profiles of chromosome 6 show higher 6q21 gain frequency in IBC tumors than in nIBC (A). Regional genomic profiles were established with CGH analytics® software (Agilent Technologies), for IBC and nIBC cases (panels B and C) both within the genomic interval [105.9–114.7 Mb] of the long arm of the chromosome 6 (hg17 human genome mapping; build 35 from NCBI). Profiles are distinguishable by different colors corresponding to different cases. Several IBC cases showed 6q21 gain or regional or focused amplification (B), whereas only two nIBC cases displayed a regional amplification (C). (PPT) [file pone.0016950.s004.ppt]
